# Integrated Analysis of Climate, Soil, Topography and Vegetative Growth in Iberian Viticultural Regions

**DOI:** 10.1371/journal.pone.0108078

**Published:** 2014-09-24

**Authors:** Helder Fraga, Aureliano C. Malheiro, José Moutinho-Pereira, Rita M. Cardoso, Pedro M. M. Soares, Javier J. Cancela, Joaquim G. Pinto, João A. Santos

**Affiliations:** 1 Centre for the Research and Technology of Agro-Environmental and Biological Sciences, Universidade de Trás-os-Montes e Alto Douro, UTAD, Vila Real, Portugal; 2 Instituto Dom Luiz, CGUL, Universidade de Lisboa, Lisbon, Portugal; 3 ADEC, Instituto Superior de Engenharia de Lisboa, Lisbon, Portugal; 4 GI-1716, Proyectos y Planificación. Dpto. Ingeniería Agroforestal, Universidad de Santiago de Compostela, Escuela Politécnica Superior, Lugo, Spain; 5 Department of Meteorology, University of Reading, Reading, United Kingdom; 6 Institute for Geophysics and Meteorology, University of Cologne, Cologne, Germany; University of Vigo, Spain

## Abstract

The Iberian viticultural regions are convened according to the Denomination of Origin (DO) and present different climates, soils, topography and management practices. All these elements influence the vegetative growth of different varieties throughout the peninsula, and are tied to grape quality and wine type. In the current study, an integrated analysis of climate, soil, topography and vegetative growth was performed for the Iberian DO regions, using state-of-the-art datasets. For climatic assessment, a categorized index, accounting for phenological/thermal development, water availability and grape ripening conditions was computed. Soil textural classes were established to distinguish soil types. Elevation and aspect (orientation) were also taken into account, as the leading topographic elements. A spectral vegetation index was used to assess grapevine vegetative growth and an integrated analysis of all variables was performed. The results showed that the integrated climate-soil-topography influence on vine performance is evident. Most Iberian vineyards are grown in temperate dry climates with loamy soils, presenting low vegetative growth. Vineyards in temperate humid conditions tend to show higher vegetative growth. Conversely, in cooler/warmer climates, lower vigour vineyards prevail and other factors, such as soil type and precipitation acquire more important roles in driving vigour. Vines in prevailing loamy soils are grown over a wide climatic diversity, suggesting that precipitation is the primary factor influencing vigour. The present assessment of *terroir* characteristics allows direct comparison among wine regions and may have great value to viticulturists, particularly under a changing climate.

## Introduction

The most renowned viticultural regions in the Iberian Peninsula (Portugal and Spain) have a long standing tradition in winemaking and are considered world-class grapevine (*Vitis vinifera* L.) producing regions. Spain, which currently has the largest vineyard area in the world (over 1×10^6^ ha), is the 3^rd^ wine producer worldwide, while Portugal ranks in the 11^th^ place, with internationally acclaimed wines, such as the Port wine [1]. In these countries, viticultural regions are convened according to Denominations of Origin (DO), or Qualified Denomination of Origin (DOCa), which are imposed by governmental institutions and controlled by strict regulations. Although the spatial distribution and limits of each DO are subjected to different rules in Portugal and Spain, the significance of the DO is nearly the same. In particular, the law enforcements are similar to both Portugal and Spain, even if they still differ in certain aspects of national legislation. The Portuguese Douro/Porto DO (henceforth Douro DO) was the first viticultural region worldwide to implement these regulations in 1756 [2]. Such regulations aim at obtaining a superior wine quality, while establishing the specific wine characteristics of each region [3]. The concept of *terroir*, which includes specific soil, topography, climate, landscape characteristics and biodiversity features of each winemaking region [4], is entrenched within the classification of a DO. Each DO is expected to be a recognized trademark, whilst other vineyards/products not included in the DO are not allowed to bear this denomination.

Being part of the *terroir*, soil is one of the most important factors for viticulture [2]. It supports the root system, which accumulates carbohydrates, absorbs water and other nutrients, being crucial for grapevine growth, physiology and yield attributes [5], [6]. Soil structure and chemistry can influence grapevine composition and consequently wine quality [7]. Compact and shallow soils can obstruct root access to oxygen, water and nutrients, limiting root growth and development [8]. In grapevines, nutrient and water uptake occur mostly within 0.5–1.0 m soil profile [2], [9]. Therefore, deep soils with good drainage (either natural or manmade) are usually preferred for vineyard installation [10]. Additionally, soil water retention properties are also important, as they can affect grapevine performance [11], [12]. A high soil water storage capacity is indeed important in regions where grapevines are subjected to excessive heat and water stress, as is the case of the Mediterranean regions [13].

Climate, also an important component of the *terroir*, is widely acknowledged as one of the most important factors for grapevine development and growth [8], [14]–[17]. During the growing season (April–October in the Northern Hemisphere), climatic conditions exert a significant influence on vine physiological processes. One of the most well-known climatic limitations of grapevine is the 10°C base temperature, needed for the onset of its yearly cycle [5]. Throughout its different stages of development, sunlight, heat and water demands vary. In fact, it has been shown that the timings and duration of the grapevine phenological stages are deeply tied to the prevailing atmospheric conditions [18], [19], which also contribute to variability in grapevine yield [20], [21], wine production [14], [22], [23] and quality [24], [25]. All these climatic factors limit the geographic distribution of grapevine [15], [26], [27], being also key factors in determining the suitability of a given region for specific varieties and wine types [27]–[29].

The topographic elements represent yet another key factor that influences viticultural and oenological characteristics of a given region. Amongst the most important topographic elements for viticulture are elevation, slope degree and aspect/exposure [11], [30]. Elevation can have a significant impact on vineyard temperatures (i.e. vertical temperature gradient), thus exerting a strong influence in site and varietal selection [2]. The slope degree of the terrain impacts on canopy microclimate (e.g. through solar exposure), soil erosion, water drainage [31] and viticultural management. The aspect refers to the compass direction to which the terrain faces (e.g. northern/southern exposure), influencing the surface net incoming solar radiation flux [31], [32], thus being determinant for site selection. These elements further enhance the singularity of viticultural regions, since they influence cultural and management practices [2]. As an example, growers tend to select row orientation according to the geographical aspect of the terrain (e.g. northern/southern exposure) in order to optimize solar radiation intercepted by the canopy. Another example is the implementation of walled terraces to overcome slope degree in steep mountainous areas, allowing mechanization and decreasing soil erosion [2].

Vine vigour is related to the growth dynamics of grapevines. It is used as an indicator of grapevine performance, affecting yield, wine production and grape quality [33]. For instance, relationships between vegetative growth and remote sensing derived metrics are broadly recognised [34]. Spectral vegetation indices have shown a good agreement with grapevine vigour, phenology, grape production and wine attributes [34]–[37], proving a suitable metric of grapevine spatial variability and performance.

The Iberian Peninsula presents a wide range of all these site-related elements influencing grapevine performance. From a climatic perspective it delivers a relatively large set of mesoclimates, spanning from dryer regions, in the inner south, to more humid regions, in the north and northwest [38], [39]. Topography and soils are also quite distinct throughout the peninsula, ranging from extended flatland areas to steep mountainous regions [40], each with very different soil characteristic [2], which may influencing crop selection and settlements in each region. All these elements are reflected in the different varieties grown throughout the peninsula [41]. Iberia presents a large number of autochthonous grapevine varieties, according to their adaptation to the different climates, soils and topographic conditions [42], with red varieties usually prevailing in the south and white ones in the north [43]. Given the heterogeneous conditions in which grapevines are grown in Iberia, understanding the complex relationships between all these factors represents a serious concern for grapevine growers and winemakers.

The present study aims to evaluate the conditions of the viticultural regions in Iberia, regarding the main features of the *terroir*. This is first integrated analysis of this kind over the entire Iberian Peninsula. Therefore, the objectives of this work are three-fold: 1) to assess the prevailing conditions in terms of climate, soil and topography in the Iberian viticultural regions; 2) to develop an integrated analysis of the previous three elements and their impact on vegetative growth; and 3) to establish a zoning of homogeneous climate-soil-topography-vegetative growth areas.

## Materials and Methods

### Viticultural regions and vineyard area

**Figure 1:**
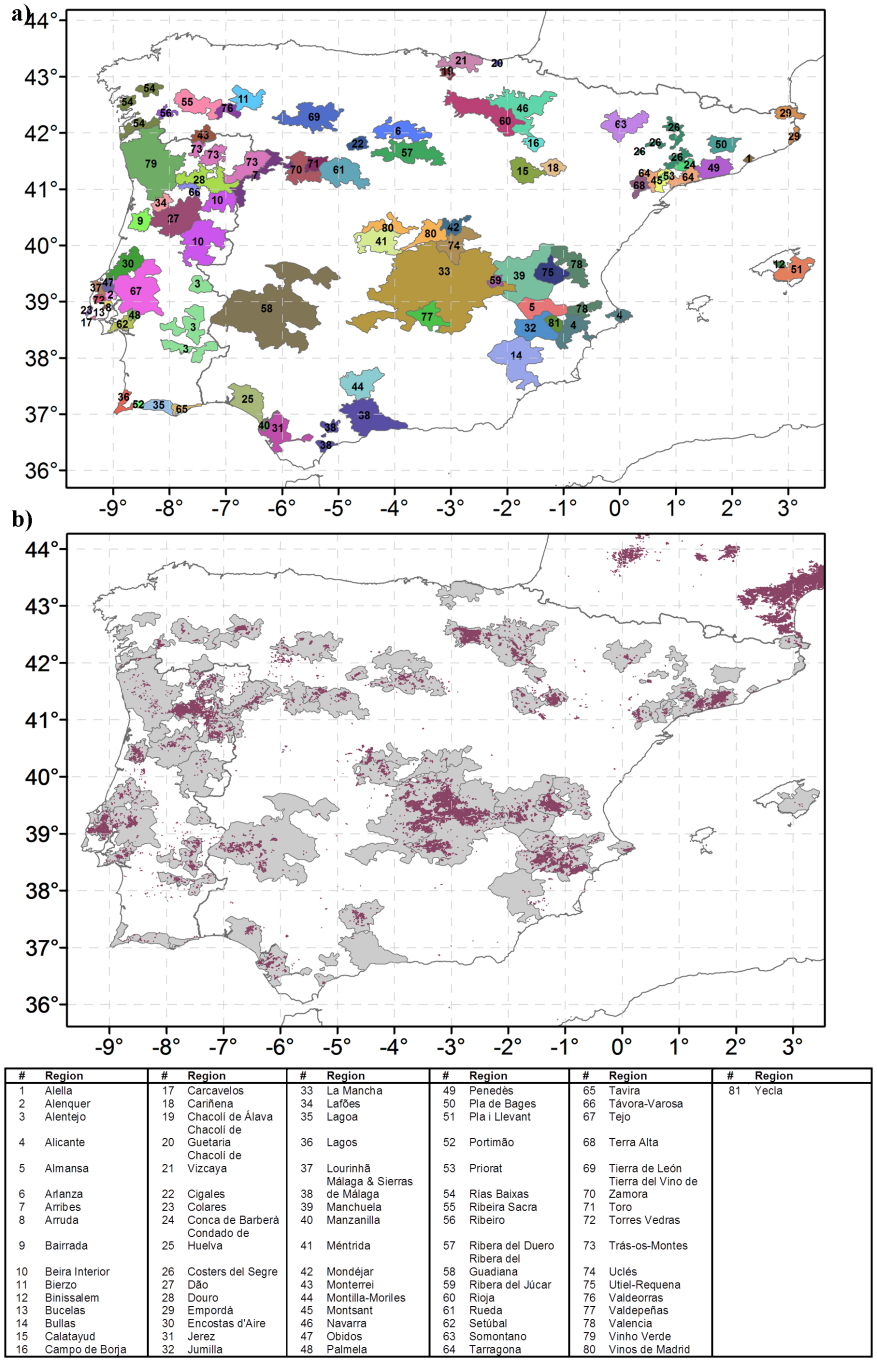
Vitcultural regions in Iberia. a) Location of the viticultural regions in Iberia, along with their denomination. b) Spatial distribution of the vineyard land cover over Iberia (dark-red), assessed using the Corine Land Cover, version 13- 2012, along with the viticultural regions (light-grey).

To assess the spatial characteristics of each DO region, boundaries of each DO or DOCa (Rioja and Priorat) were defined using data available in the Portuguese *‘Instituto do Vinho e da Vinha’* (IVV; http://www.ivv.min-agricultura.pt) and Spanish ‘*Ministerio de Agricultura, Alimentación y Medio Ambiente’* (MAGRAMA; http://sig.magrama.es). The viticultural regions in the islands of Madeira, Azores (Portugal) and Canarias (Spain) were not assessed due to limitations in the soil and climatic datasets. Other viticultural regulated regions, such as, quality wine with specific geographical indication, estate wine, qualified estate wine and country wines, Indicação de Proveniencia Regulamentada (in Spain) and Vinho Regional (in Portugal), are out of the scope of the current study, since DO regions are usually considered of higher importance. Note that the DO regions of Málaga and Sierras de Málaga (in Spain) are two different DO regions that geographically coincide and were therefore treated jointly (henceforth DO Málaga & Sierras de Málaga). As a result, the spatial boundaries of 81 DO regions (82 effectively), 25 in Portugal and 56 (57) in Spain, were identified within Iberian Peninsula (Fig. 1a).

In order to analyse the spatial distribution of the vineyards in Iberia, the Corine Land Cover Map, version 13–2012, was used [44], [45]. This dataset, last updated in 2012, provides an inventory of the land cover over Europe and has previously proven to have high accuracy in representing the land cover over mainland Portugal and Spain [46], [47]. The vineyard fraction of the land cover over Iberia was extracted from this dataset for subsequent analysis (Fig. 1b).

### Topography

For the topographic analysis, elevation and geographical aspect were selected as the leading topographic elements to be studied, due to their importance in Iberian viticulture. Although slope degree (degree of inclination of the land) is also considered a key landscape element, it was not included in the current analysis, as in Iberian vineyards certain practices, such as walled terraces, are often implemented to flatten steep-slopes. As detailed information of such practices was not available, this cannot be taken into account. For the elevation assessment, the GTOPO30 digital elevation model (DEM) was used at the 30 arc-seconds spatial resolution (https://lta.cr.usgs.gov/GTOPO30). Elevation ranges are isolated inside each region (according to Fig. 1a, b). Aspect was calculated using the same GTOPO30 DEM and geographical information systems.

### Climate

In order to analyse the climatic conditions of each region, a categorized bioclimatic index for viticultural zoning (CatI) was used [29]. CatI establishes climatic categories by combining three bioclimatic indices (Table 1): Huglin Index [48], Dryness Index [49] and Cool Night Index [50]. The Huglin Index expresses the thermal potential of a given region and relates the heat accumulation to the physiological development of grapevines. The Dryness Index assesses water availability for grapevines, by estimating potential water balance over the growing season. The Cool Night Index accounts for minimum temperatures at the end of the vegetative cycle (September in the Northern Hemisphere), as lower nocturnal temperatures during this stage tend to be favourable for wine quality [50]. Thus, CatI allows determining the optimum climatic suitability in terms of phenological development, heat and water availability conditions during the growth season, as well as ripening conditions (Table S1).

**Table 1:** Categorized Index (CatI), along with the corresponding classes of the combined indices: Huglin, Dryness and Cool Night indices, according to [29].

CatI	Huglin Index (°C) [48]	Dryness Index (mm) [49]	Cool Night Index (°C) [50]	Description
0	<900	<−100		Unsuitably cold or excessively dry
1	900 – 1500	−100 – 50	<14	Cool, dry with cool nights
2	900 – 1500	−100 – 50	> 14	Cool, dry with warm nights
3	900 – 1500	> 50	<14	Cool, humid with cool nights
4	900 – 1500	> 50	> 14	Cool, humid with warm nights
5	1500 – 2100	−100 – 50	<14	Temperate, dry with cool nights
6	1500 – 2100	−100 – 50	> 14	Temperate, dry with warm nights
7	1500 – 2100	> 50	<14	Temperate, humid with cool nights
8	1500 – 2100	> 50	> 14	Temperate, humid with warm nights
9	2100 – 2700	−100 – 50	<14	Warm, dry with cool nights
10	2100 – 2700	−100 – 50	> 14	Warm, dry with warm nights
11	2100 – 2700	> 50	<14	Warm, humid with cool nights
12	2100 – 2700	> 50	> 14	Warm, humid with warm nights
13	> 2700	−100 – 50	<14	Very warm, dry with cool nights
14	> 2700	−100 – 50	> 14	Very warm, dry with warm nights
15	> 2700	> 50	<14	Very warm, humid with cool nights
16	> 2700	> 50	> 14	Very warm, humid with warm nights

For CatI calculation (and combining indices), data from a regional climate model (RCM) Weather Research and Forecast model (WRF) [51] version 3.1.1 was used. The WRF model is a non-hydrostatic model and has been widely used for dynamical downscaling regional climate. For the present climate, a simulation with a horizontal grid resolution of 9-km (nested in a 27-km grid) was used, with both grids centred in the Iberian Peninsula. The RCM simulation started at 00 00 UTC 1 January 1989 and ended at 18 00 UTC 31 January 2013, with initial, lateral and lower boundary conditions derived from ERA-Interim. From the model output, precipitation and temperature over Iberia were considered for this study. A more detailed description of the model set-up can be found in Soares et al. [52] and Cardoso et al. [53], where the simulation results were validated for inland maximum and minimum temperatures and precipitation, showing a good agreement with observations. Patterns of the simulated CatI were then compared to previously established patterns using observational data [29], showing a good agreement. Additionally, solar radiation (surface net downward shortwave flux), from the Modern Era Retrospective-analysis for Research and Applications (MERRA; http://gmao.gsfc.nasa.gov/merra/) at a 0.6º×0.6º longitude/latitude spatial resolution, was also obtained. Mean values over the growing season were calculated for 1989–2012.

### Soils

For soil analysis, the predominant soil texture (SoilT) was assessed according to the United States Department of Agriculture soil textural classification [54]. The texture of a soil refers to its relative content of clay, sand and silt particles (Table 2). Each soil texture class presents its own properties in terms agricultural applicability. Clay soils have fine particles and retain large amounts of water, but are poorly drained and usually difficult to manage [55], [56]. Conversely, sandy soils are coarse and usually excessively drained, with low water retention capacity [55], [56]. With relatively even proportions between particles, the loamy soils are typically well drained and provide sufficient nutrient retention and are thus usually preferable for agricultural use [55], [56]. Soil texture is a fundamental soil property used as a qualitative classification tool to determine other soils properties [54], such as soil plasticity, drainage and available water content [56]. Soil texture classes (SoilT) were obtained from the Harmonized World Soil Database (HWSD) [57].

**Table 2:** Soil texture categories, along with the respective percentages of Clay, Silt and Sand, according to USDA soil textural classification [54].

Soil Category	Clay (%)	Silt (%)	Sand (%)	Texture
1	60–100	0–40	0–45	heavy clay
2	40–60	40–60	0–20	silty clay
3	40–60	40–60	0–45	clay
4	27–40	40–73	0–20	silty clay loam
5	27–40	15–52	20–45	clay loam
6	0–12	88–100	0–20	silt
7	0–27	74–88	20–50	silty loam
8	35–55	0–20	45–65	sandy clay
9	7–27	28–50	23–52	loam
10	20–35	0–28	45–80	sandy clay loam
11	0–20	0–50	50–70	sandy loam
12	0–15	0–30	70–86	loamy sand
13	0–10	0–14	86–100	sand

### Vegetative growth

The Enhanced Vegetation Index (EVI) was used for the analysis of the grapevine vigour. Spectral vegetation indices are based on visible and near-infrared radiation fluxes, captured by sensors on-board of polar orbiting satellites, and are a measure of the concentration of green leaf vegetation in space [58]. The EVI algorithm accounts for canopy background (e.g. soil and bare earth) and atmospheric effects (e.g. clouds), while also being barely affected by manmade structures [58], [59].

In this study, the EVI from the Moderate Resolution Imaging Spectroradiometer (MODIS - MOD13A2 Collection 5) was extracted from the National Aeronautic and Space Administration (NASA) Land Processes Distributed Active Archive Center (LP DAAC; https://lpdaac.usgs.gov/). The EVI is described through the following equation (Eq. 1):
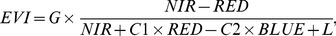
(Eq.1)


where NIR is the near-infrared band (841–876 nm), RED is the red band (620–670 nm), BLUE is the blue band (459–479 nm), L is the canopy background coefficient (L = 1), C1 and C2 are aerosol resistance and influence coefficients of the blue and red bands, respectively (C1 = 6 and C2 = 7.5), and G is a gain factor (G = 2.5) [60], [61]. Four MODIS tiles (h17v04, h17v05, h18v04 and h18v05), covering all of the Iberian mainland were obtained at a 1-km spatial resolution for the 2012 growing season. This single year was selected to match the latest available land cover update (described in section 2.1), which ensures land cover changes do not interfere with the analysis of grapevine vegetative growth. The mean growing season EVI was then calculated (April-October mean) and the spatial average for the vineyard areas over Iberia was 0.23. For categorization of each DO according to the vegetative growth, two EVI classes (EVIc-1 and 2) were defined: EVIc-1 (EVI ≤ 0.23), for low vegetative growth areas, and EVIc-2 (EVI> 0.23), for high vegetative growth.

## Results

### Mesoscale patterns

**Figure 2:**
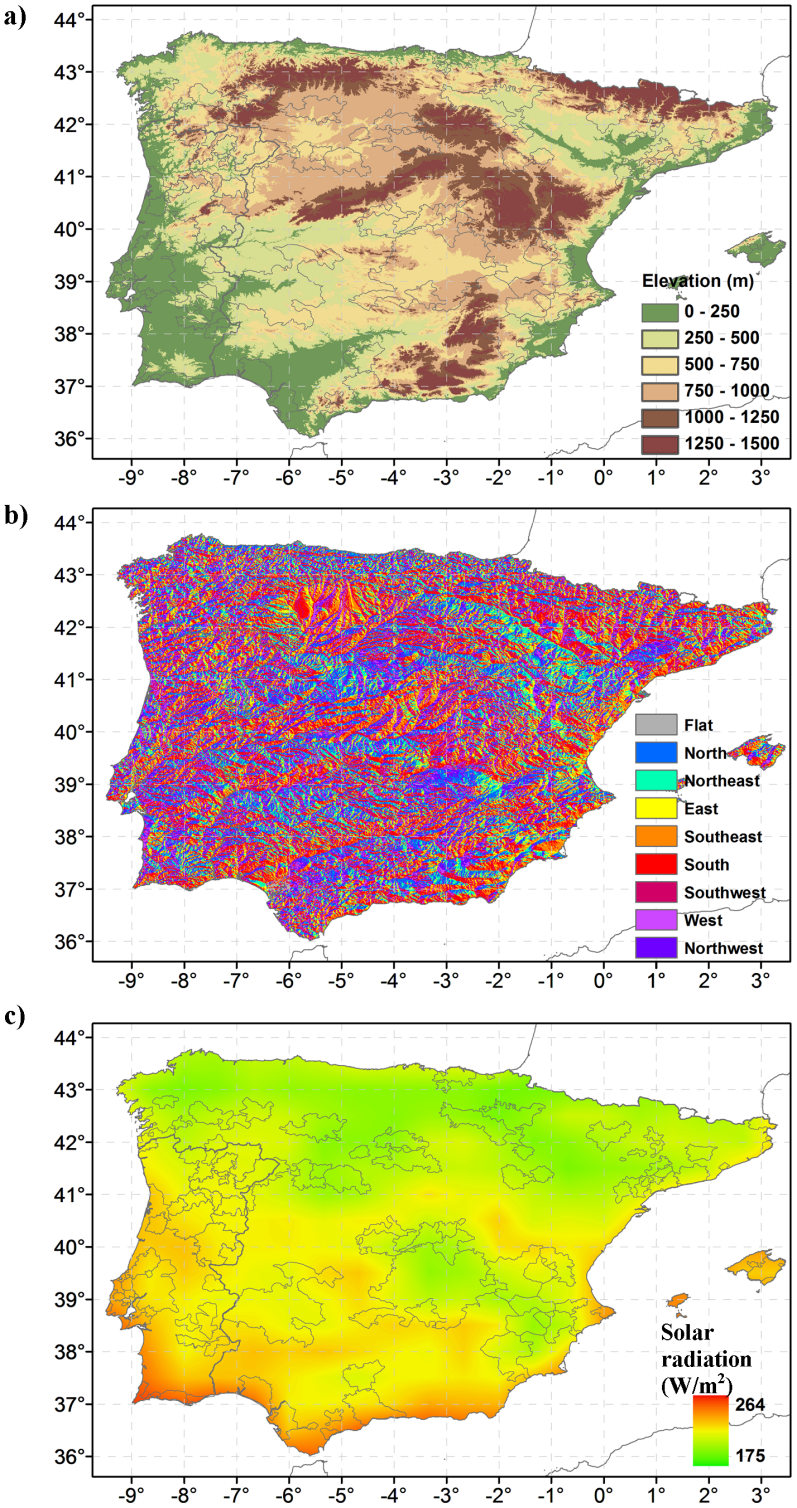
The topography in Iberia. a) Elevation (m) in the Iberian Peninsula, calculated using the GTOPO30 dataset. b) As in (a) but for the aspect. c) Solar radiation over Iberia, mean growing season values in 1989–2012 calculated using MERRA data at a 0.6ª spatial resolution.

Overall, topography over Iberia displays large differences in both elevation and aspect (Fig 2a, b). Effective solar radiation depicts a strong north/south contrast, with higher solar radiation values in the south (Fig. 2c). Noticeable is the relatively low solar radiation in centre western Iberia (near La Mancha DO, #33), when compared to the surrounding areas. Grapevines in these areas are generally less affected by excessive solar radiation.

**Figure 3:**
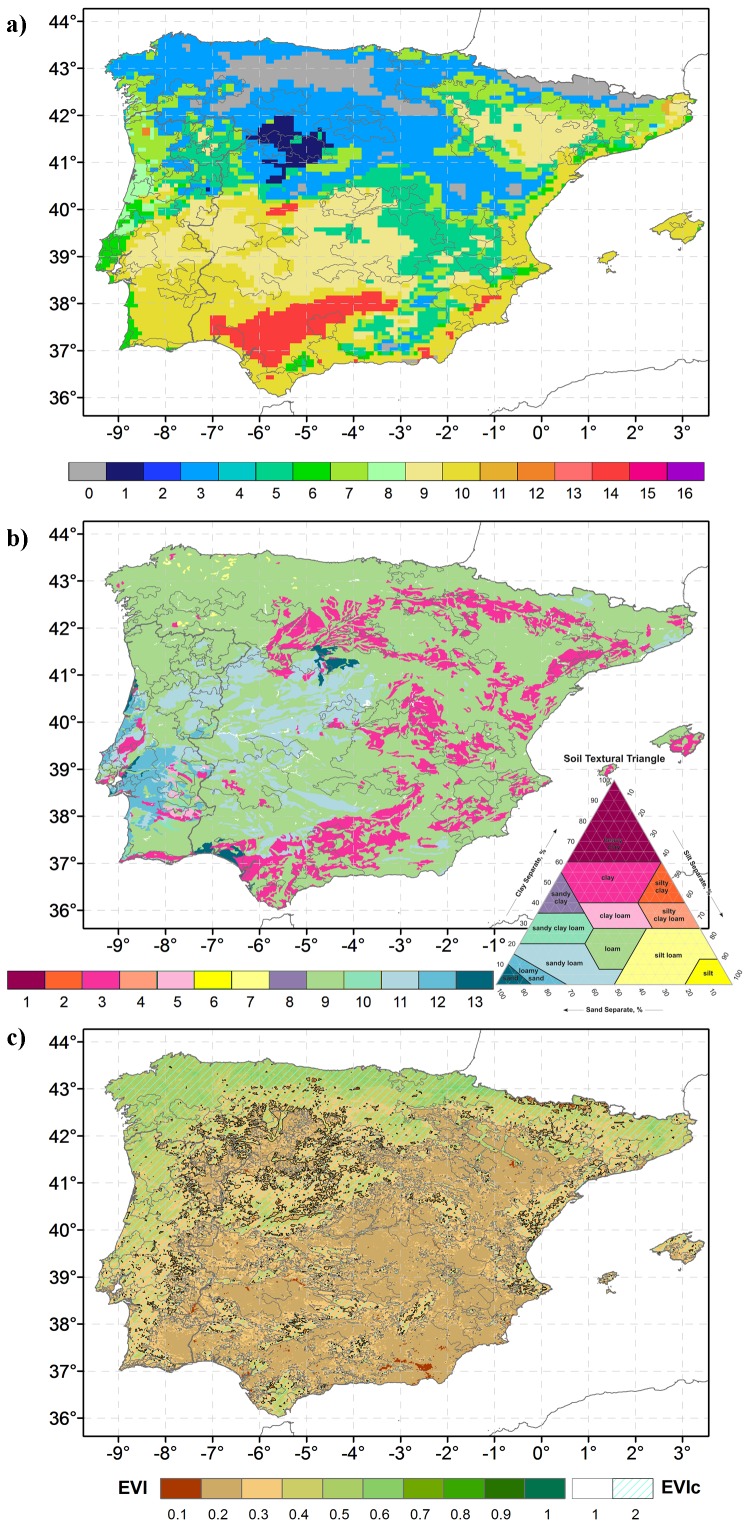
Climate, soil and vegetative growth in Iberia. a) CatI over Iberia calculated according to Table 1, for the period of 1989-2012 using WRF simulations. b) SoilT according to Table 2 using HWSD data. c) Mean EVI and EVIc for the grapevine growth period (April-October) in 2012, using MODIS data. The spatial-average of the vineyard areas corresponds to 0.23. Below this value the EVIc equals 1 (transparent overlay), above this value EVIc equals 2 (hatched overlay).

CatI (Fig. 3a) depicts a clear distinction between the northern regions, generally cooler, and the southern regions, with higher thermal accumulation. In fact, most of northern Iberia is situated in CatI-3 (cool, humid, with cool nights), making this the predominant category regarding all of Iberia. Also in the north, CatI-0 climates are present, suggesting lack of viticultural suitability owing to insufficient thermal accumulation. The lower category, CatI-1 (Cool, dry, with cool nights), is observed in an isolated area near the centre of the peninsula. Conversely, the warmer climatic region CatI-14 is located in the centre-south. CatI-10/9 (warm, dry, with warm/cool nights), represent the 2^nd^/3^rd^ dominant climatic categories, scattered across the south and northeast. CatI-5 (Temperate, dry with cool nights) occurs over a large area in centre/northern Iberia, being the 4^th^ dominant category (taking into account all of the mesoscale pattern in Iberia). Other secondary categories appear in transitional areas, between cool and warm climates. For example, CatI-11/12, with warm and humid climates, appear isolated in opposite sides of the peninsula (east/west), depicting the singularity of these regions.

Soil textural classes present a very homogeneous pattern (Fig. 3b and Table 2). Loamy soils (SoilT-9), which are commonly considered highly suitable for agriculture, are prevalent in most of the Iberian Peninsula. However, in central Iberia, sandy-loam soils (SoilT-11) are more frequent, whereas in southwestern Iberia loamy-sand soils (SoilT-12) are also common. Clay loam soils (SoilT-5) are isolated in a small region in southwestern Iberia. Clay soils (SoilT-3) dominate most of southern and eastern Iberia. In northwest Iberia, some intrusions of silt-loam soil (SoilT-7) are also depicted.

Regarding the vegetative development, represented by EVI and EVIc (Fig. 3c), the north/south and west/east contrasts are clear. Northern and western areas generally present a much higher EVI than southern and eastern areas. This was particularly clear in the EVIc, where the coastal areas in northern and western Iberia are classified as EVIc-2 (high vigour), while the rest of Iberia was generally keyed to EVIc-1 (low vigour).

Spearman ranked correlation coefficients between the previous mesoscale patterns (Table S2) reveal weak to moderate correlations. The highest positive correlation was found between CatI and surface net solar radiation flux (0.61), as both indirectly reflect the latitudinal effect over temperature and incoming solar radiation. The strongest negative correlation (−0.66) found was between CatI and elevation, undoubtedly reflecting the temperature lapse rate already embedded into this climatic index. A negative moderate correlation (−0.35) found was between EVI and CatI, while a weak positive correlation was found between EVI and SoilT (0.12).

### Integrated analysis

**Figure 4:**
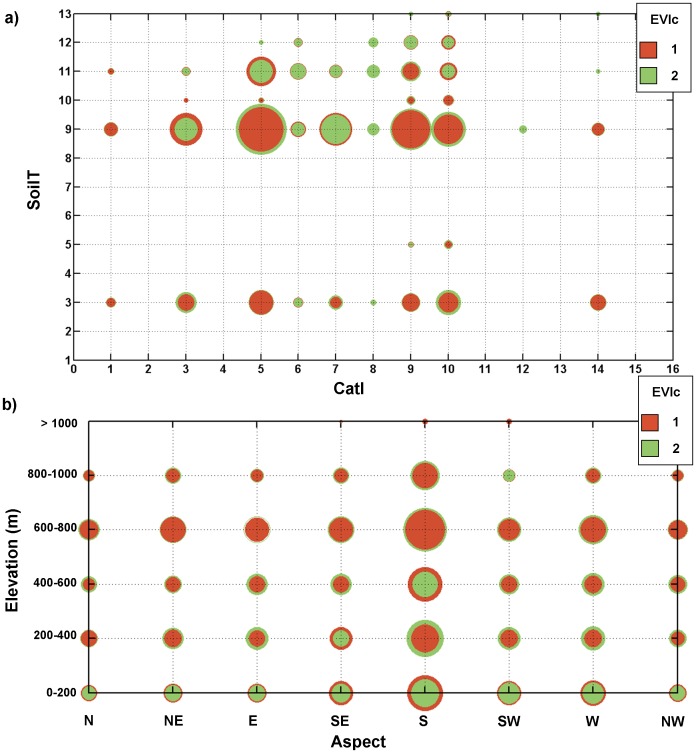
Integrated analysis. a) Circular accumulated EVIc (1 – red, 2 – green) as a function of the SoilT and CatI for all vineyards in Iberia. b) Circular accumulated EVIc as a function of the elevation and aspect for all vineyards in Iberia. The size of each circular chart depicts the accumulated vineyard area belonging to that EVI class, and the inner (outer) circular class depicts the largest (smallest) EVI class.

An integrated analysis of the selected *terroir* elements was performed taking only the grapevine growing areas into account (Fig. 4). Fig. 4a depicts the grapevine vegetative growth in Iberia (EVIc) as a function of their climates (CatI) and soil characteristics (SoilT). Nearly all vineyards situated in SoilT-3 (clay) tend to show lower vegetative growth (EVIc-1), whereas higher vigour (EVIc-2) prevails in SoilT-11/12 (Warm, humid with cool/warm nights). Much more diverse vigour conditions are keyed to SoilT-9 (loam), which is the most frequent soil type in Iberia (Fig. 4a). In this soil type, vineyards show either high vigour (EVIc-2), in regions with temperate climates (CatI-6, 7 and 8), or low vigour (EVIc-1), in regions with cool (CatI-1 to 4) or warm (CatI-9 to 16) climates (Fig. 4a). However, there are some exceptions: in SoilT-9 and CatI-5 (temperate climate) vineyards depict low vigour, while for SoilT-9 (loam) under cool/warm climates high vigour only occurs when combined with humid conditions. Thus, the influence of dryness/humidity (a CatI component) on SoilT-9 vineyards is highlighted for cool/warm conditions, which was not verified in other soil types.

Concerning the climatic influences on vegetative growth, in cool climatic regions (CatI-1 to 4) low vigour (EVIc-1) prevails, with the exception of the more humid regions (CatI-3) with loam/sandy-loam soils (SoilT-9/11). On the other hand, CatI-6 and 8 regions (Temperate, warm nights and dry or humid respectively) predominantly show high vigour (EVIc-2) (Fig. 4a), regardless of SoilT. The same applies to CatI-7 vineyards, with the exception of SoilT-3 areas. For warmer regions (CatI-9, 10, 12 and 14), low vigour is also dominant, apart from sandy loam or loamy sand soils (SoilT-11/12), which mostly have high vigour vineyards.

The same integrated analysis was also performed taking into account grapevine vegetative growth as a function of elevation and aspect (Fig. 4b). The largest vineyard concentrations are located at 600–800 m elevation ranges. Low elevation vineyards (most of them along coastal strips) present much higher vegetative growths, which can be partly explained by the more humid coastal local climates, or by the existence of deeper soils in these regions. Regarding the geographical aspect, growers tend to prefer south-facing vineyards, but no strong relationship can be established between aspect and vegetative growth.

### DO regional analysis

With respect to vineyard locations (Fig. 1b), they are mostly confined by the DO boundaries, with the largest visible concentrations of vineyards in La Mancha and Rioja (Spain, #33 and #60) and Douro (Portugal, #28). Overall, 81 DO are depicted in Fig. 1a. These regions show large differences in terms of shape and area (Fig.1a; Table S3). The largest DO is La Mancha (Spain) and the smallest is Carcavelos (Portugal, #17).

**Figure 5:**
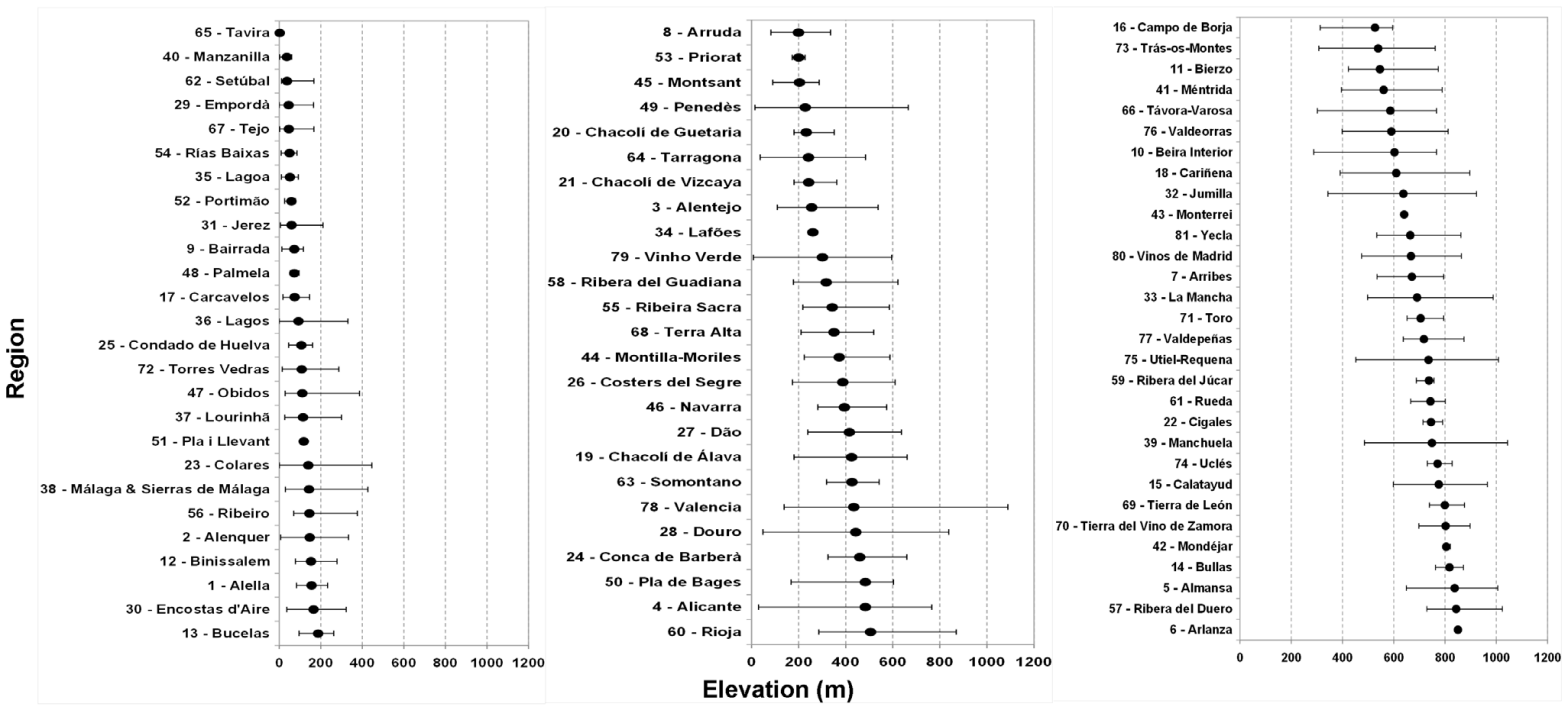
Elevation (m) of the vineyards in each DO/DOCa in Iberia. The inner circle represents the mean elevation and the horizontal bars represent the minimum and maximum, of the locations of the vineyards inside the DO.

The elevation means and ranges of the vineyard areas for each DO (Fig. 5) highlight the large spatial variability in which vineyards are grown, ranging from near sea-level (e.g. DO Tavira, #65) to elevations over 850 m (DO Arlanza, #6). Near coastal regions tend to have vineyards at much lower mean elevations, while in the innermost DO regions vineyards show higher mean elevations and ranges (Fig. 2a). The largest vineyard areas are indeed located at mean elevations ranging from 600 to 800 m, with the most heterogeneous being DO Valencia, which is also one of largest in terms of surface (Table S3). As expected, small DO regions, such as Tavira (#65), Pla i Llevant (#51), Lafões (#34), Monterrei (#43), Mondéjar (#42) and Arlanza (#6), tend to show much lower spatial variability. It should be noticed that La Mancha (with the largest vineyard area; Table S3) shows relatively low variability in elevation (relatively flat area) when compared to other smaller regions in mountainous areas (e.g. Douro, Table S3).

**Figure 6:**
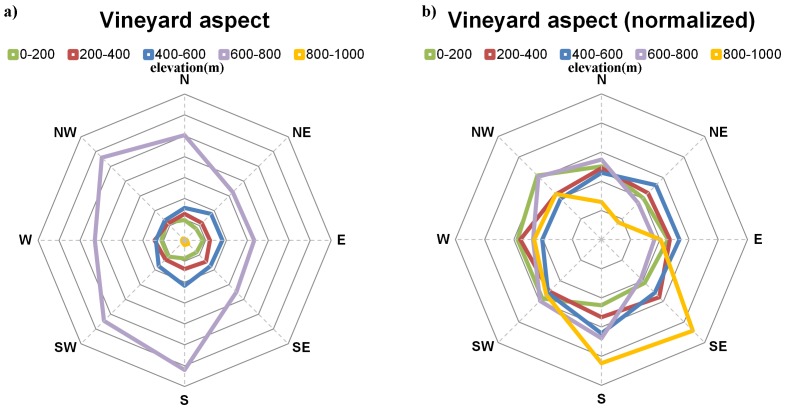
Vineyard aspect in Iberia. a) Geographical aspect (orientation) vineyards in Iberia according to the mean elevation of each DO. b) as in (a) but normalized using the vineyard area.

When analysing the geographical aspect of the vineyards within each DO (Fig. 6), it is clear that terrains with an S-SW aspect are preferred for viticultural activities. This outcome mainly reflects the largest vineyard area located at 600–800 m elevations (Fig. 6a). Despite this fact, vineyards in 800–1000 m elevation tend to have S-SE aspects (Fig. 6b), while vineyards at lower elevations (0–200 m) have N-NW aspects (Fig. 6b). For elevations in the 200–600 m range, no clear distinction on aspect preference can be made. Regarding the solar radiation, while most DO regions in Portugal present a high solar radiation, DO regions in Spain are usually located in areas with lower radiations (with the exception of some regions in the south).

In Table 3, the 81 DO are defined according to their predominant categories of CatI, SoilT and EVIc. The cool, dry, with cool nights DO regions (CatI-1) of Tierra del Vino de Zamora (#70) and Toro (#71) exhibit similar clay soils (SoilT-3) with low vegetative growth (EVIc-1), while the also CatI-1 DO Rueda (#61) exhibits sandy-loam soils (SoilT-11) and high vegetative growth (EVIc-2). This effect is less visible in the equally cool, but more humid, CatI-3 regions of Ribera del Duero (#57), Arlanza (#6), Cigales (#22), Chacolí de Álava (#19), Chacolí de Guetaria (#20), Tierra de León (#69) and Valdeorras (#76). While in the first two regions, SoilT-3 (clay) is predominant, each one shows a different EVIc (1 and 2; low and high vigour respectively). The other CatI-3 regions depict SoilT-9 (loam) and EVIc-2, with the exception of Cigales that exhibits EVIc-1.

**Table 3:** CatI, SoilT and EVI class for each viticultural region in Iberia.

#	Region	CatI	SoilT	EVIc	#	Region	CatI	SoilT	EVIc	#	Region	CatI	SoilT	EVIc
70	Tierra del Vino de Zamora	1	3	1	1	Alella	6	9	2	10	Beira Interior	9	11	2
71	Toro	1	3	1	2	Alenquer	6	9	2	67	Tejo	9	12	1
61	Rueda	1	11	2	8	Arruda	6	9	2	48	Palmela	9	12	2
57	Ribera del Duero	3	3	1	23	Colares	6	9	2	35	Lagoa	10	3	1
6	Arlanza	3	3	2	45	Montsant	6	9	2	44	Montilla-Moriles	10	3	1
22	Cigales	3	9	1	13	Bucelas	6	11	2	68	Terra Alta	10	3	1
19	Chacolí de Álava	3	9	2	47	Obidos	6	11	2	38	Málaga & Sierras de Málaga	10	3	2
20	Chacolí de Guetaria	3	9	2	72	Torres Vedras	6	11	2	65	Tavira	10	3	2
69	Tierra de León	3	9	2	37	Lourinhã	6	12	2	3	Alentejo	10	5	2
76	Valdeorras	3	9	2	60	Rioja	7	3	1	12	Binissalem	10	9	1
14	Bullas	5	3	1	63	Somontano	7	3	1	52	Portimão	10	9	1
24	Conca de Barberà	5	3	1	16	Campo de Borja	7	9	1	78	Valencia	10	9	1
39	Manchuela	5	3	1	11	Bierzo	7	9	2	29	Empordà	10	9	2
59	Ribera del Júcar	5	3	1	21	Chacolí de Vizcaya	7	9	2	51	Pla i Llevant	10	9	2
42	Mondéjar	5	3	2	34	Lafões	7	9	2	64	Tarragona	10	9	2
49	Penedès	5	3	2	43	Monterrei	7	9	2	62	Setúbal	10	12	2
4	Alicante	5	9	1	54	Rías Baixas	7	9	2	40	Manzanilla	10	13	1
5	Almansa	5	9	1	55	Ribeira Sacra	7	9	2	25	Condado de Huelva	10	13	2
18	Cariñena	5	9	1	56	Ribeiro	7	9	2	31	Jerez	14	3	1
28	Douro	5	9	1	79	Vinho Verde	7	9	2					
53	Priorat	5	9	1	30	Encostas d'Aire	8	11	2					
73	Trás-os-Montes	5	9	1	9	Bairrada	8	12	2					
74	Uclés	5	9	1	33	La Mancha	9	3	1					
75	Utiel-Requena	5	9	1	32	Jumilla	9	9	1					
81	Yecla	5	9	1	46	Navarra	9	9	1					
15	Calatayud	5	9	2	58	Ribera del Guadiana	9	9	1					
66	Távora-Varosa	5	9	2	77	Valdepeñas	9	9	1					
7	Arribes	5	11	1	80	Vinos de Madrid	9	9	1					
27	Dão	5	11	2	26	Costers del Segre	9	9	2					
17	Carcavelos	6	3	1	50	Pla de Bages	9	9	2					
36	Lagos	6	3	1	41	Méntrida	9	11	1					

Only the predominant categories are shown.

As previously mentioned, CatI-5 (Temperate, dry, with cool nights) was the 4^th^ leading category (regarding all Iberia). Nonetheless, it is the dominant category when only the area under vineyards is considered, being present in 19 DO regions (Bullas (#14), Conca de Barberà (#24), Manchuela (#39), Ribera del Júcar (#59), Mondéjar (#42), Penedès (#49), Alicante (#4), Almansa (#5), Cariñena (#18), Douro (#28), Priorat (#53), Trás-os-Montes (#73), Uclés (#74), Utiel-Requena (#75), Yecla (#81), Calatayud (#15), Távora-Varosa (#66), Arribes (#7) and Dão (#27)). These regions have SoilT-3, 9 and 11. For CatI-5, only SoilT-11 seems to present higher vegetative growth (in more than 50% of these DO regions). However, for CatI-6 regions (same as CatI-5 but with warm nights), all SoilT-9, 11 and 12 regions are keyed to EVIc-2 (Alella (#1), Alenquer (#2), Arruda (#8), Colares (#23), Montsant (#45), Bucelas (#13), Obidos (#47), Torres Vedras (#72) and Lourinha (#37).

The CatI-7 (Temperate, humid with cool nights) regions show the same relationship with soil as previously seen for CatI-3. Since these regions present humid conditions, SoilT-9 (loam) is clearly beneficial for vegetative growth when compared to SoilT-3 (clay). CatI-8 (Temperate, humid with warm nights) regions (Encostas d'Aire (#30) and Bairrada (#9)) are the only regions where EVIc-2 (high vigour) is present, regardless of SoilT. CatI-9 and CatI-10 (warm, dry with cool or warm nights respectively) regions display similar characteristics to those already reported, higher vegetative growths in SoilT-9, 11 and 12 than in SoilT-3. The only DO regions in Iberia that currently present very warm, dry, with warm nights climate (CatI-14) is the DO Jerez (#31), that also exhibits clay soils (SoilT-3) and consequently low vegetative growth (EVIc-1).

## Discussion and Conclusions

In the current study, an integrated analysis of the climate, soil, topography and vegetative growth was undertaken for the Iberian viticultural areas, using state-of-the-art datasets. Until present, studies on viticultural zoning were mainly focused on climatic conditions [15], [62]–[64], while the combination of the *terroir* composing elements was still underexplored. While in the last decades great advances have been made regarding the quality and availability of these spatial datasets [65], few studies have been devoted to integrate these factors into viticultural zoning [11], [66], [67], but none for Iberia. Therefore, understanding the spatial variability of these factors provides the basis for a viable characterization of each viticultural region. To our knowledge this is the first study in which climate, soil, topography and vegetative growth, were jointly studied to analyse the viticultural regions in the Iberian Peninsula.

Overall, temperate dry climate with cool nights (CatI-5) is the dominant climatic category for vineyards in Iberia. Further, the results showed that vineyards in DO regions with CatI-5 tend to present lower vigour. Nevertheless, these conditions of moderate water restriction and low nocturnal temperatures during ripening are often beneficial for the production of high quality wines [14], [24], [68], possibly explaining the higher vineyard density in these regions. Other temperate climate types (CatI-6, 7 and 8) tend to present higher levels of vigour, regardless of soil type, topography or dryness levels (CatI component). This may be partially explained by the fact that grapevines tend to be less exposed to heat and water stress under these climatic conditions, thus experiencing significantly fewer restrictions to its development and growth. Reversely, in warmer and cooler climates, abiotic constrains significantly increase, enhancing the importance of other factors, such as precipitation and soil type, for vine performance [31], [69]–[72].

The results suggested that soil also plays a key role in viticulture, as regions with similar climatic conditions, but with different soil types, can indeed present different vigour attributes. Soils with higher clay content are associated with lower vigour. This can be explained by water uptake restrictions, due to lower root penetration and stronger soil-water retention [73]. Depending on their structure, clay soils can also promote high vigour [74], though not apparent in the present study. To further investigate this, a more detailed soil study including soil structure, porosity and depth would be required, which was not the object of this study. On the other hand, soils with lower clay and higher sand contents seem to promote higher vigour. Although sandy soils retain less water than clay soils, they are better drained, providing a better growing structure for roots and higher absorption capacity for water and mineral nutrients. The results also showed that loamy soils are the most common type in Iberian vineyards, where precipitation plays a leading role in governing the vigour patterns, since more humid (dry) regions tend to present higher (lower) vigour (Fig. 4a). These outcomes enhanced the importance of soil texture in determining vine performance in each region.

Regarding the topographic features, a clear distinction was found between low and high elevations, with the former showing higher vigour vineyards. However, this could be an indirect manifestation of climate-elevation relationships, such as the temperature lapse rate, the Atlantic influence in the Iberian Mediterranean-like climates (higher precipitation amounts over northern and western Iberia than over central and eastern Iberia [14]) and/or the presence of deeper soils with higher water holding capacity in low-elevation areas. For the geographical aspect and considering the peninsula as a whole, no clear relationship with vegetative growth was found, despite some regional differences.

Based on previous research some considerations can still be made on the effects of the main components of the *terroir* in wine quality. First, it has been shown that climate plays a key role on wine quality [2], [5]. Regions with temperate/temperate-warm dry climates with cool nights (in this study CatI-5/9) tend to produce a more balanced maturation, by e.g. promoting the synthesis of anthocyanins, resulting in higher quality. Second, soil type is also an important factor for quality, but its suitability largely depends on the targeted wine typicity and attributes. Previous studies showed that wines from clay soils (SoilT-3) showed high sugar accumulation and anthocyanin concentration [66], [73], but lower pH [7], than those from sandy soils (SoilT-11/12) [66], while other studies suggest that loamy soils (SoilT-9) may also be favourable to high quality wines [75], [76]. Third, studies have shown that lower vigour vineyards (EVIc-1) are generally expected to produce higher grape and wine quality, with higher total soluble solids, total phenolics and anthocyanins and lower titratable acidity [34], but are commonly associated with lower yield [77], [78]. From the present study results, some considerations can also be made regarding the regional quality attributes. As an example, the renowned Douro/Porto DO shows climate (CatI-5; Temperate, dry with cool nights), soil (SoilT-9; Loamy) and vigour (EVIc-1; low) conditions particularly suitable for high quality wine production [2], [5]. These conditions are also shared by other regions, such as Alicante, Almansa, Cariñena, Priorat, Trás-os-Montes, Uclés, Utiel-Requena and Yecla, highlighting the potential for the future viticultural development of these regions. However, the Iberian Peninsula presents a wide range of *terroirs* (Table 3), from e.g. Toro (CatI-1, SoilT-3 and EVIc-1) to Jerez (CatI-14, SoilT-3 and EVIc-1) or Setúbal (CatI-10, SoilT-12 and EVIc-2), and in all cases these regions are known to produce high quality wines but with different typicity. Nonetheless, it should be noted that the present study did not directly include other important *terroir* elements, such as viticultural and oenological practices, which are also key for the wine attributes produced in each region.

The assessments provided herein may be of great value to viticulturists and may also play a key role when including vineyards into a given DO. Usual methods for DO delineation rely on onsite analysis of climate, soil and topographic attributes. Although these empirical approaches are extremely useful, they are often based on erratic, insufficient or unreliable data (e.g. outdated land cover, soils surveys and topographic maps, assessments made on nearby weather station records), making comparison between regions rather difficult. Furthermore, taking into account the climate change projections for Iberia [15], [17], [29], [79], [80], these delineations may require and more continuous update. Our fully integrated approach provides a feasible method for DO comparison on a mesoscale basis. This may allow growers to identify new management practices and grapevine varieties that can be easily adapted to other regions that share the same *terroir* characteristics. Additionally, the implemented methodologies can be extended to other viticultural regions of the world.

## Supporting Information

Table S1 **The Cool Nigh, Dryness and Huglin indices, along with their mathematical definition, units and classes.**
(DOCX)

Table S2 **Spearman ranked correlation coefficient between the EVI, CatI, SoilT, elevation, aspect and solar radiation in all of Iberia.**
(DOCX)

Table S3 **Area (km^2^) of each DO region in Iberia.**
(DOCX)
