# Supplementary material for: Integrated Analysis of Climate, Soil, Topography and Vegetative Growth in Iberian Viticultural Regions
Source: PLoS One. 2014 Sep 24;9(9):e108078. doi: 10.1371/journal.pone.0108078 (PMC4176712; doi:10.1371/journal.pone.0108078)
Supplement: Table S1 — The Cool Nigh, Dryness and Huglin indices, along with their mathematical definition, units and classes. (DOCX) [file pone.0108078.s001.docx]

**Table S1 -** The Cool Nigh, Dryness and Huglin indices, along with their mathematical definition, units and classes.

| **Index** | **Mathematical definition** | **Units** | **Classes** |
| --- | --- | --- | --- |
| **Cool Night Index (CI)** | September average *Tmin* (°C)  (North hemisphere) | °C | Very cool nights: < 12  Cool nights: 12 – 14  Temperate nights: 14 – 18  Warm nights: > 18 |
| **Dryness Index (DI)** | $\sum_{April}^{Sept.} (Wo+P-Tv-Es)$  Wo - Initial available soil water reserve (mm) on the first month / DI on the following months;  P – Precipitation (mm);  T_v_ - Potential vineyard transpiration (mm);  E_s_ - Direct evaporation from the soil (mm)  T_v_; E_s_ are assessed using the Thornthwaite method | mm | Excessively dry: <-100  Moderately dry: -100 – 50  Sub-Humid: 50 – 150  Humid: > 150 |
| **Huglin Index (HI)** | $\sum_{April}^{Sept.} \frac{(T-10)+(T_{\max}-10)}{2}d$  T - Mean air temperature (°C);  T_max_ - Maximum air temperature (°C);  d - Length of day coefficient, from 1.02 to 1.06 | °C | Unsuitably Cool: < 900  Too Cool: 900 – 1200  Very Cool: 1200 – 1500  Cool: 1500 – 1800  Temperate: 1800 – 2100  Warm/Temperate: 2100 – 2400  Warm: 2400 – 2700  Very Warm: 2700 – 3000  Too Hot: > 3000 |
