# Supplementary material for: Integrated Analysis of Climate, Soil, Topography and Vegetative Growth in Iberian Viticultural Regions
Source: PLoS One. 2014 Sep 24;9(9):e108078. doi: 10.1371/journal.pone.0108078 (PMC4176712; doi:10.1371/journal.pone.0108078)
Supplement: Table S2 — Spearman ranked correlation coefficient between the EVI, CatI, SoilT, elevation, aspect and solar radiation in all of Iberia. (DOCX) [file pone.0108078.s002.docx]

**Table S2 -** Spearman ranked correlation coefficient between the EVI, CatI, SoilT, elevation, aspect and solar radiation in all of Iberia.

|  | EVI | CatI | SoilT | Elevation | Aspect | Radiation |
| --- | --- | --- | --- | --- | --- | --- |
| EVI |  |  |  |  |  |  |
| CatI | -0.35 |  |  |  |  |  |
| SoilT | 0.12 | 0.04 |  |  |  |  |
| Elevation | 0.02 | -0.66 | -0.10 |  |  |  |
| Aspect | 0.01 | 0.01 | 0.03 | -0.01 |  |  |
| Radiation | -0.29 | 0.61 | 0.13 | -0.28 | -0.01 |  |
