# Supplementary material for: Integrated Analysis of Climate, Soil, Topography and Vegetative Growth in Iberian Viticultural Regions
Source: PLoS One. 2014 Sep 24;9(9):e108078. doi: 10.1371/journal.pone.0108078 (PMC4176712; doi:10.1371/journal.pone.0108078)
Supplement: Table S3 — Area (km2) of each DO region in Iberia. (DOCX) [file pone.0108078.s003.docx]

**Table S3 -** Area (km^2^) of each DO region in Iberia.

| **#** | **Region** | **Area (km^2^)** | **#** | **Region** | **Area (km^2^)** | **#** | **Region** | **Area (km^2^)** |
| --- | --- | --- | --- | --- | --- | --- | --- | --- |
| 1 | Alella | 140 | 32 | Jumilla | 2507 | 63 | Somontano | 2261 |
| 2 | Alenquer | 270 | 33 | La Mancha | 23569 | 64 | Tarragona | 1623 |
| 3 | Alentejo | 4873 | 34 | Lafões | 693 | 65 | Tavira | 454 |
| 4 | Alicante | 2645 | 35 | Lagoa | 987 | 66 | Távora-Varosa | 419 |
| 5 | Almansa | 2069 | 36 | Lagos | 608 | 67 | Tejo | 5459 |
| 6 | Arlanza | 2231 | 37 | Lourinhã | 345 | 68 | Terra Alta | 766 |
| 7 | Arribes | 1780 | 38 | Málaga & Sierras de Málaga | 5184 | 69 | Tierra de León | 3294 |
| 8 | Arruda | 128 | 39 | Manchuela | 6059 | 70 | Tierra del Vino de Zamora | 1828 |
| 9 | Bairrada | 1034 | 40 | Manzanilla | 196 | 71 | Toro | 719 |
| 10 | Beira Interior | 5639 | 41 | Méntrida | 2555 | 72 | Torres Vedras | 273 |
| 11 | Bierzo | 1497 | 42 | Mondéjar | 926 | 73 | Trás-os-Montes | 3566 |
| 12 | Binissalem | 156 | 43 | Monterrei | 772 | 74 | Uclés | 2103 |
| 13 | Bucelas | 46 | 44 | Montilla-Moriles | 2667 | 75 | Utiel-Requena | 1775 |
| 14 | Bullas | 5012 | 45 | Montsant | 593 | 76 | Valdeorras | 670 |
| 15 | Calatayud | 1608 | 46 | Navarra | 3755 | 77 | Valdepeñas | 1789 |
| 16 | Campo de Borja | 624 | 47 | Obidos | 437 | 78 | Valencia | 3432 |
| 17 | Carcavelos | 38 | 48 | Palmela | 295 | 79 | Vinho Verde | 7926 |
| 18 | Cariñena | 829 | 49 | Penedès | 1653 | 80 | Vinos de Madrid | 3054 |
| 19 | Chacolí de Álava | 324 | 50 | Pla de Bages | 1121 | 81 | Yecla | 630 |
| 20 | Chacolí de Guetaria | 88 | 51 | Pla i Llevant | 1782 |  |  |  |
| 21 | Chacolí de Vizcaya | 1517 | 52 | Portimão | 177 |  |  |  |
| 22 | Cigales | 625 | 53 | Priorat | 180 |  |  |  |
| 23 | Colares | 118 | 54 | Rías Baixas | 2065 |  |  |  |
| 24 | Conca de Barberà | 427 | 55 | Ribeira Sacra | 2120 |  |  |  |
| 25 | Condado de Huelva | 2638 | 56 | Ribeiro | 400 |  |  |  |
| 26 | Costers del Segre | 1791 | 57 | Ribera del Duero | 3125 |  |  |  |
| 27 | Dão | 3859 | 58 | Ribera del Guadiana | 16353 |  |  |  |
| 28 | Douro | 2555 | 59 | Ribera del Júcar | 415 |  |  |  |
| 29 | Empordà | 1070 | 60 | Rioja | 3715 |  |  |  |
| 30 | Encostas d'Aire | 1819 | 61 | Rueda | 2846 |  |  |  |
| 31 | Jerez | 2409 | 62 | Setúbal | 724 |  |  |  |
